# Trends of improved water and sanitation coverage around the globe between 1990 and 2010: inequality among countries and performance of official development assistance

**DOI:** 10.1080/16549716.2017.1327170

**Published:** 2017-06-12

**Authors:** Seungman Cha, Paul Mansiangi Mankadi, Mousab Siddig Elhag, Yongjoo Lee, Yan Jin

**Affiliations:** ^a^ Department of Disease Control, Faculty of Infectious and Tropical Disease, London School of Hygiene & Tropical Medicine, London, UK; ^b^ Environmental Health Department, School of Public Health, University of Kinshasa, Kinshasa, Democratic Republic of the Congo; ^c^ Communicable and Non-Communicable Diseases Control Directorate, General Directorate of Primary Health Care, Federal Ministry of Health, Khartoum, Sudan; ^d^ Executive Director, Team & Team International, Seoul, Republic of Korea; ^e^ Department of Microbiology, Dongguk University College of Medicine, Gyeongju, Republic of Korea

**Keywords:** Improved water and sanitation, inequality, official development assistance targeting, performance, trend

## Abstract

**Background**: As the Millennium Development Goals ended, and were replaced by the Sustainable Development Goals, efforts have been made to evaluate the achievements and performance of official development assistance (ODA) in the health sector.

In this study, we explore trends in the expansion of water and sanitation coverage in developing countries and the performance of ODA.

**Design**: We explored inequality across developing countries by income level, and investigated how ODA for water and sanitation was committed by country, region, and income level. Changes in inequality were tested via slope changes by investigating the interaction of year and income level with a likelihood ratio test. A random effects model was applied according to the results of the Hausman test.

**Results**: The slope of the linear trend between economic level and sanitation coverage has declined over time. However, a random effects model suggested that the change in slope across years was not significant (e.g. for the slope change between 2000 and 2010: likelihood ratio χ^2^ = 2.49, probability > χ^2^ = 0.1146). A similar pro-rich pattern across developing countries and a non-significant change in the slope associated with different economic levels were demonstrated for water coverage. Our analysis shows that the inequality of water and sanitation coverage among countries across the world has not been addressed effectively during the past decade. Our findings demonstrate that the countries with the least coverage persistently received far less ODA per capita than did countries with much more extensive water and sanitation coverage, suggesting that ODA for water and sanitation is poorly targeted.

**Conclusion**: The most deprived countries should receive more attention for water and sanitation improvements from the world health community. A strong political commitment to ODA targeting the countries with the least coverage is needed at the global level.

## Background

Access to safe and clean water and sanitation has been explicitly recognized as a human right and as essential for the full enjoyment of life by the United Nations (UN) [1]. There have been remarkable achievements in increasing the extent of improved water and sanitation coverage over the last two decades [2]. More than 2.6 billion people have gained access to improved water since 1990, and 2.1 billion to improved sanitation [2].

However, there is still room for improvement. Unsafe water and unsafe sanitation were the 14th and 19th leading risk factors of global disability-adjusted life years for both sexes in 2015 [3], and inadequate access to water, sanitation, and hygiene practices (WASH) was found to cause 58% of diarrhoeal deaths [4]. Improved water and sanitation are essential for eliminating neglected tropical diseases [5,6]. Globally, approximately 2.4 billion people live without any improved sanitation facility inside their household compound, and 663 million people do not have access to improved drinking water sources [2].

There have been global efforts to increase improved water and sanitation coverage after the Millennium Declaration. A substantial increase in official development assistance (ODA) in the WASH sector has coincided with the implementation of the Millennium Development Goals (MDGs) [7].

ODA is defined as government aid designed to promote the economic development and welfare of developing countries by the Organisation for Economic Co-operation and Development (OECD). A list of developing countries and territories is maintained by the OECD, and ODA is counted as only aid to these countries. ODA does not include loans and credits for military purposes.

Improved water coverage has rapidly increased since 2000 because of the global efforts. The MDG target of halving the proportion of the population without sustainable access to safe drinking water was met in 2010, five years ahead of the original 2015 deadline [8,9].

However, we don’t know whether such progress has been made in the countries with the least coverage, or if the pro-rich pattern of WASH coverage among developing countries has changed.

In this study, we explored the trends of water and sanitation improvements across developing countries and the performance of ODA, with a particular focus on inequality among countries and ODA targeting. According to the World Health Organization (WHO), health inequalities are defined as differences in health status or in the distribution of health determinants between different population groups [10]. We created an operational definition for this study, according to which water and sanitation inequality were defined as differences in water and sanitation coverage between different population groups.

Inequality has become a main issue in health in general [11] and for access to WASH in particular [2]. Universal health coverage is the ultimate goal for the health-related Sustainable Development Goals (SDGs) and emphasizes reducing inequality within and among countries [12,13].

However, studies on inequality have largely been country-level analyses. Even studies [14,15] analysing a large number of countries have focused on inequality in individual countries rather than exploring inequalities among countries. It is well understood and reported that tremendous pro-rich inequalities exist in improved water and sanitation coverage between the rich and poor within a country [2], but we do not know how unequally improved water and sanitation coverage is distributed among developing countries by wealth level. Pro-rich inequality refers to the unequal distribution of coverage, with higher coverage among wealthier population groups. We believe that it will be important to design global policy to investigate inequality among developing countries, since investigations limited in scope to the country level are insufficient to provide the global development community with guidance related to global priorities.

In this study, we explored inequality in improved water and sanitation coverage by income level among developing countries across the world and the trend of inequality between 1990 and 2010. In addition, we assessed whether increases of improved water and sanitation coverage have been larger in countries with more needs. We investigated the distribution of ODA for water and sanitation during this period and evaluated whether ODA has targeted the countries with the greatest needs. Finally, we estimated how many child deaths will be averted during the SDG period by expanding improved water and sanitation coverage at the national and global levels.

There has been extensive debate among the authors about the appropriate target period for this study; in particular, we had to make a difficult choice between 1990–2010 and 1990–2015.

This study examines trends over two decades rather than the status at a specific time point, and in particular, we were concerned about whether the trends of WASH had changed after the worldwide MDGs campaign was proclaimed. We thus found it adequate to investigate the trend change between the decade before the Millennium Declaration (1990–2000) and the decade afterwards (2000–2010), so that we could evaluate whether any change in the trends took place between the same absolute time periods. Therefore, we concluded that 2010 was a more important time point than 2015 for developing counter-arguments against the ‘success story’ of WASH coverage. Ultimately, we decided to choose 1990–2010 for this study. To our knowledge, although similar studies have been conducted previously in the maternal and child health sector, this is the first in the WASH sector.

## Methods

### Data sources

We used the OECD database for the years from 2001 through 2010. Commitment data were obtained from the OECD’s Creditor Reporting System (CRS) database [16]. The coverage of water and sanitation in 1990–2010 was obtained from the World Health Statistics of the WHO [17]. Population-related data for the years from 2001 through 2010, including the total number of neonatal deaths and the total population, were taken from the *State of The World’s Children* publications of UNICEF [18–27]. The CRS database is publicly available and provides data that have been updated since 9 April 2010. We used ODA commitments from all channels (public sector, non-governmental organizations and civil society, public–private partnerships, multilateral organizations, and others) and all donors (Development Assistance Committee [DAC] countries, multilateral, non-DAC countries, and private donors such as the Bill and Melinda Gates Foundation), and current prices (US$) were applied.

### Data analysis

#### Inequality of water and sanitation coverage among countries by income level

In order to explore inequality across developing countries by the level of wealth, we calculated absolute measures of inequality [28] in water and sanitation coverage. If all groups were to have presented the same increase during 2000–2010, the absolute measures of inequality would have remained unchanged. The slope index of inequality was not used because the deprivation level of each income group could not be identified across the developing countries.

Changes in inequality were tested via slope changes by investigating the interaction of year and income level with a likelihood ratio test. A random effects model was applied according to the results of the Hausman test. Stata version 12 (Stata Corporation, College Station, TX, USA) was used for the analysis.

#### ODA trends

In order to compare the total amount of ODA by income group, we extracted data from 181 countries: 55 least developed countries, including 6 others in the low-income group; 40 lower-middle-income countries; 54 upper-middle-income countries; and 32 more advanced developing countries and territories by income level.

Of these countries, we chose 103 countries for further detailed analysis. These countries were identified as those where the under-five mortality rate was above 40 per 1000 live births in 1990, the baseline year of the MDGs. This originally resulted in 105 countries, but Sudan and South Sudan were excluded due to the absence of data. While investigating the targeting of ODA towards the countries with the highest burdens, we paid particular attention to the 5 countries (China, Democratic Republic of the Congo, India, Nigeria, and Pakistan; hereafter called ‘the Big 5’) where the greatest number of children die [29], the 15 countries (Afghanistan, Angola, Burkina Faso, China, DR Congo, Ethiopia, India, Indonesia, Kenya, Mali, Niger, Nigeria, Pakistan, Tanzania, and Uganda; hereafter called ‘the Big 15’) where 75% of pneumonia- and diarrhoea-specific child deaths take place [30], and the 70 ‘Countdown’ countries, where more than 95% of maternal and child deaths occur [31]. We investigated how ODA for water and sanitation was committed by country, region, and income level.

#### Effectiveness of coverage increases and ODA targeting

This study explored whether the coverage increase for water and sanitation was larger in the countries with the greatest needs than in other countries, and examined whether ODA for water and sanitation has targeted the countries with the poorest coverage. In our analysis, the need for water and sanitation was defined in terms of the coverage of water or sanitation in the baseline year of any comparison. We used methodologies presented in previous studies [32,33], particularly for investigating ODA targeting and trends therein.

We investigated whether increases in water coverage were correlated with increases in sanitation coverage, and whether ODA commitment per capita was associated with the real increases in the coverage of water and sanitation in recipient countries.

#### Number of under-five child deaths to be averted in 2016–2030

We estimated the number of under-five deaths that could be prevented by expanding water and sanitation coverage up to 90% by 2030 in each country, with a constant rate of annual increase. To estimate the effect of scaling up sanitation and water coverage on the health of children, we used the Lives Saved Tool (LiST). This model estimates the effect of scaling up interventions on maternal and child health. Further details on LiST are presented in the Discussion section.

## Results

### Inequality among developing countries by economic level

**Figure 1.**
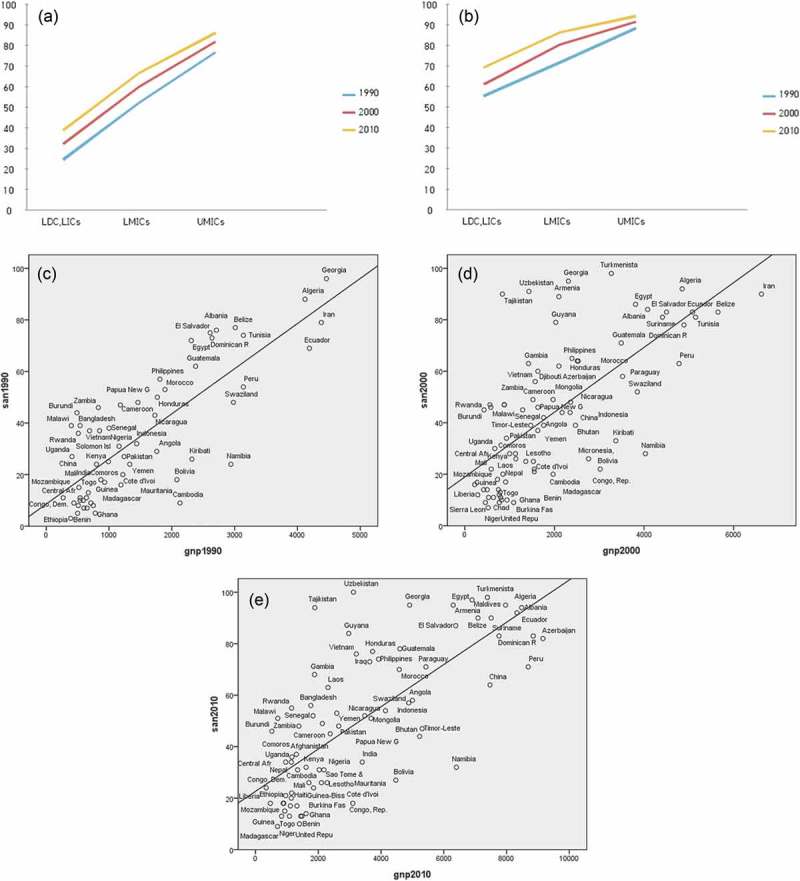
Improved sanitation/water coverage by gross national product (GNP) per capita in 1990, 2000, 2010. (a) Trend change for sanitation coverage between countries grouped by income (X-axis: country group by income level, Y-axis: sanitation coverage (%)). (b) Trend change for water coverage between countries grouped by income (X-axis: country group by income level, Y-axis: water coverage (%)). (c) Improvements in sanitation coverage by per capita GNP in 1990. (d) Improvements in sanitation coverage by per capita GNP in 2000. (e) Improvements in sanitation coverage by per capita GNP in 2010. Notes: LDC, least developed country; LIC, low-income country; LMIC, lower-middle-income country; UMIC, upper-middle-income country.

Water and sanitation coverage showed a consistently pro-rich pattern among developing countries (Table 1, Figure 1). For example, as the economic level shifted by one category across developing countries in 1990, sanitation coverage differed by 23.98% (95% confidence interval [CI], 20.21–27.75%). Moreover, the coverage increase associated with a one-unit increase in the economic level across developing countries dwindled to 20.68% (95% CI, 17.03–24.36%) in 2010. The slope of the linear trend has declined over time.

**Table 1.** Inequality in water and sanitation coverage among developing countries by economic level.

Intervention	Year	Regression coefficient^a^	95% CI	*p*-value	R^2^	Likelihood ratio
Water	1990	14.46	11.67, 17.25	< 0.001	0.4594	
	2000	13.06	10.74, 15.39	< 0.001	0.4597	
	2010	10.47	8.20, 12.75	< 0.001	0.3885	
	Slope change between 1990 and 2000			0.4395		0.60
	Slope change between 2000 and 2010			0.1146		2.49
Sanitation	1990	23.98	20.21, 27.75	< 0.001	0.5822	
	2000	22.48	19.04, 25.92	< 0.001	0.5383	
	2010	20.68	17.03, 24.36	< 0.001	0.482	0.34
	Slope change between 1990 and 2000			0.5581		0.34
	Slope change between 2000 and 2010			0.4803		0.50

Notes: ^a^Regression model; dependent variable: water or sanitation coverage, independent variable: income level.

However, a random effects model suggested that the change in slope across years was not significant (e.g. for the slope change between 2000 and 2010: likelihood ratio χ^2^ = 2.49, probability > χ^2^ = 0.1146). A similar pro-rich pattern across developing countries and a non-significant change in the slope associated with different economic levels were demonstrated for water coverage, as shown in Table 1 and Figure 1.

### Coverage increase by need (defined in terms of coverage in the baseline year)

**Figure 2.**
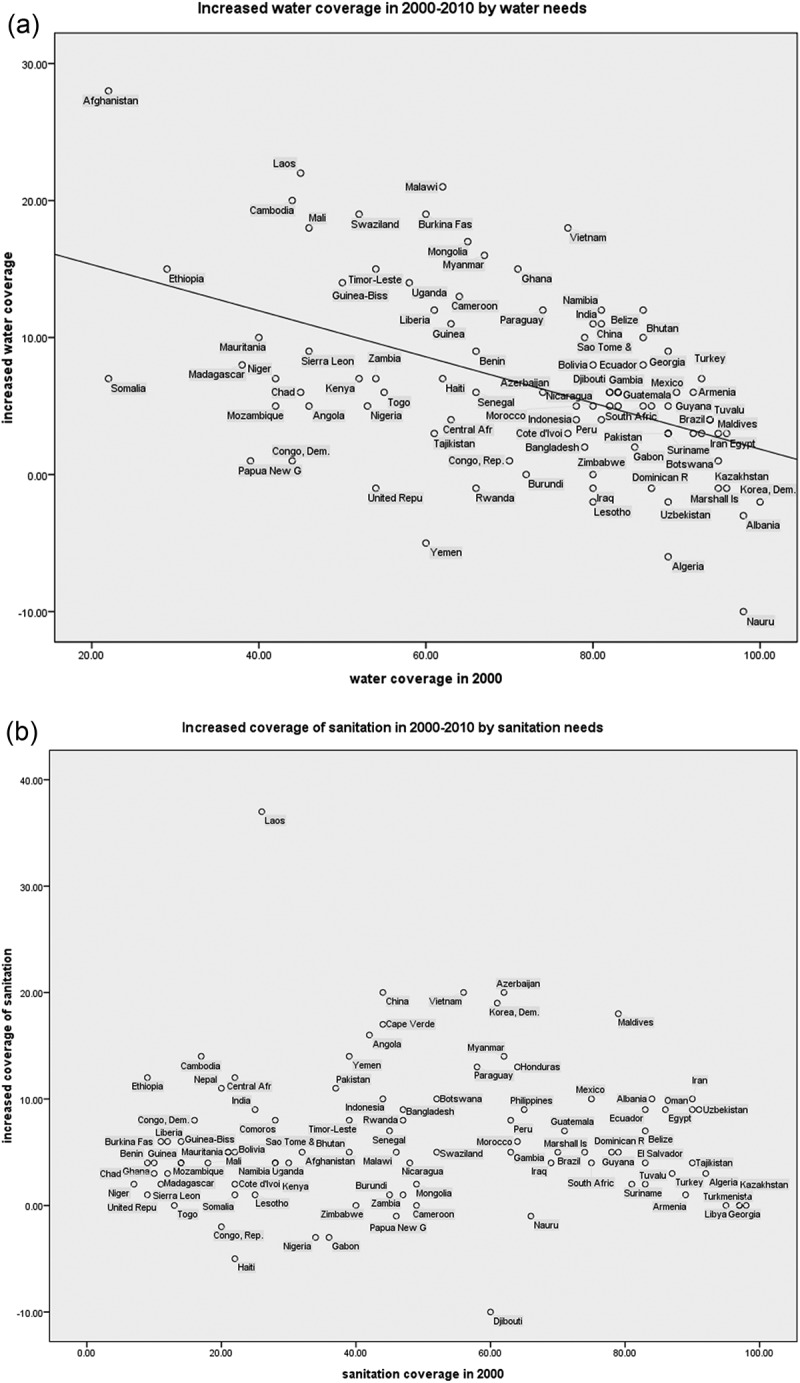
(a) Increased water coverage 2000–2010 by water needs. (b) Increased coverage of sanitation 2000–2010 by sanitation needs.

The coverage of water and sanitation during this period is shown in Table 2 and Figure 2. Figure 2 and Table 2 show whether water or sanitation coverage increased in the countries with the greatest needs.

**Table 2.** Official Development Assistance (ODA) performance in water and sanitation.

Regression model					
Independent	Dependent	β coefficient	R^2^	*p*-value	Study topic
Water coverage in 2000	Increased water coverage, 2000–2010	–0.168	0.227	< 0.001	Increase depending on need
Water coverage in 1990	Increased water coverage, 1990–2000	–0.216	0.131	< 0.001	
Increased water coverage, 2000–2010	Increased sanitation coverage, 2000–2010	0.200	0.032	0.046	Integration of water and sanitation
Correlation			Coefficient	*p*-value	
Sanitation coverage in 2000	Increased sanitation coverage, 2000–2010			0.860	Increase depending on need
ODA per capita per decade	Increased water coverage, 2000–2010			0.317	ODA contribution
ODA per capita per decade	Increased sanitation coverage, 2000–2010			0.491	
ODA per capita per decade	Increased water coverage, 2000–2010		0.205	0.039	
Water coverage in 2000	ODA per capita, 2000–2010		0.205	0.037	ODA targeting
Sanitation coverage in 2000	ODA per capita, 2000–2010			0.165	
Water coverage in 2000	ODA per capita in 2001			0.958	
Water coverage in 2000	ODA per capita in 2002			0.540	ODA targeting on a yearly basis
Water coverage in 2000	ODA per capita in 2003			0.661	
Sanitation coverage in 2000	ODA per capita in 2001			0.396	
Sanitation coverage in 2000	ODA per capita in 2002			0.521	
Sanitation coverage in 2000	ODA per capita in 2003			0.786	

Note: ODA, official development assistance.

For water, increase of coverage was correlated with need during 1990–2010. If the water coverage of a country was 1% lower than any other country in 1990, coverage in that country increased by 0.22% during 1990–2000, suggesting that countries with low water coverage had a more rapid increase. This correlation was also observed during 2000–2010, but the linear slope of coverage increase depending on need became flatter than the line for the previous decade, suggesting that the comparative speed of the increase in the countries with low coverage decreased in 2000–2010. For sanitation, however, the increase of coverage was not associated with need at the baseline year in either period, suggesting that sanitation did not increase more rapidly in the countries with more desperate needs.

### ODA targeting in the water and sanitation sector

The results for ODA targeting in the water and sanitation sector are shown in Table 2.

A correlation was found between need (using coverage as a proxy indicator of need) and the total amount of ODA per capita committed to water during the first decade of the MDGs period.

For sanitation, no association was observed between ODA per capita and need during the 10-year period. These results suggest that ODA for sanitation poorly targeted the countries with the least coverage.

Table 2 shows that the total increase of water or sanitation coverage was not associated with the total amount of ODA commitment to water and sanitation during the 10-year period in the Countdown countries, suggesting that the increases in water and sanitation coverage across the Countdown countries were not principally due to ODA.

Increases in water coverage were correlated with increases in sanitation coverage, but the correlation was not strong and the linear trend was weak (R^2^ = 0.032). We infer that increases in water and sanitation coverage might have not been implemented in a highly integrated way, although doing so has been strongly recommended [34].

#### Poor coverage in the Big 15 countries

Of note, the Big 15 countries, where 75% of pneumonia- and diarrhoea-specific under-five mortality occurs, retained low coverage of both water and sanitation, and this was a more serious problem for sanitation. The coverage in the Big 15 countries was 33% for sanitation and 65.40% for water, whilst the mean of the 103 countries analysed in this study was 54.58% for sanitation and 78.28% for water. Niger had only 9% coverage for sanitation and Tanzania, Burkina Faso, Ethiopia, Mali, and DR Congo had less than 30% coverage. Ethiopia, DR Congo, and Niger had less than 50% water coverage (Table 3).

**Table 3.** Coverage and progress of improved water and sanitation in the main countries with high child mortality.

	Sanitation						Water					
		Coverage (%)			Increase (%)			Coverage (%)			Increase (%)	
		1990	2000	2010	1990–2000	2000–2010		1990	2000	2010	1990–2000	2000–2010
Total (103)	Mean	42.79	47.95	54.58	13.32	6.21	Mean	65.99	71.48	78.28	15.69	5.55
	SD	28.11	27.56	28.40	23.68	6.45	SD	20.77	18.92	16.95	26.95	11.87
Big 5	Mean	23.00	31.20	40.20	8.20	9.00	Mean	62.60	69.40	75.60	6.80	6.20
	SD	10.42	10.89	15.91	8.23	8.22	SD	16.70	19.65	22.48	5.81	4.60
Big 15	Mean	19.00	25.73	33.00	8.00	7.27	Mean	49.07	55.60	65.40	9.80	9.80
	SD	11.15	13.29	16.87	8.72	5.96	SD	18.66	19.36	17.76	6.90	7.87
Rank												
1	Niger	5	7	9	2	2	Ethiopia	14	29	44	15	15
2	Tanzania	7	9	10	2	1	Congo, DR	45	44	45	–1	1
3	Burkina Faso	8	11	17	3	6	Niger	35	42	49	7	7
4	Ethiopia	3	9	21	6	12	Afghanistan	No data	22	50	22	28
5	Mali	15	18	22	3	4	Angola	42	46	51	4	5
6	Congo, DR	9	16	24	7	8	Tanzania	55	54	53	–1	–1
7	Nigeria	37	34	31	–3	–3	Nigeria	47	53	58	6	5
8	Kenya	25	28	32	3	4	Kenya	44	52	59	8	7
9	India	18	25	34	7	9	Mali	28	46	64	18	18
10	Uganda	27	30	34	3	4	Uganda	43	58	72	15	14
11	Afghanistan	No data	32	37	32	5	Burkina Faso	43	60	79	17	19
12	Pakistan	27	37	48	10	11	Indonesia	70	78	82	8	4
13	Indonesia	32	44	54	12	10	China	67	80	91	13	11
14	Angola	29	42	58	13	16	India	69	81	92	12	11
15	China	24	44	64	20	20	Pakistan	85	89	92	4	3

Notes: Ranked in order of coverage from lowest to highest. ODA, official development assistance.

#### Total and per capita ODA commitment to water and sanitation

During 2000–2010, US$ 59.82 billion (current price) was committed in support of water supply and sanitation in all developing countries. As a group, US$ 37.02 billion was committed to the 70 Countdown countries during this period, representing 61.88% of all water supply and sanitation commitments. US$ 9.94 billion (16.63%) was committed to the Big 5 countries, and US$ 17.69 billion (29.57%) to the Big 15 countries. Overall, ODA for water supply and sanitation tended to increase after 2002, with a mean annual change of 12.21%, corresponding to US$ 4.03 million (standard deviation, US$ 7.23 million). In 2001–2010, the largest amount of ODA for water supply and sanitation was committed to India, accounting for 9.67% of the total amount, followed by Iraq (9.56%) and Vietnam (7.54%), and the mean annual change was highest in Iraq (Table 4).

**Table 4.** Top 10 countries receiving the most Official Development Assistance (ODA) for water and sanitation.

	Country	Year												
Rank		2001	2002	2003	2004	2005	2006	2007	2008	2009	2010	Total	%	Mean annual change (%)
1	India	249.13	154.82	278.82	101.96	586.25	646.18	914.90	308.52	803.89	291.72	4336.19	9.67	59.58
2	Iraq	0.49	0.95	47.38	887.84	741.48	675.70	207.53	704.09	459.88	562.34	4287.68	9.56	765.50
3	Vietnam	279.86	201.06	229.45	297.84	194.32	433.87	192.26	541.54	653.56	355.36	3379.12	7.54	22.82
4	China	208.94	203.52	228.12	168.92	559.06	244.48	555.91	87.18	159.03	162.27	2577.44	5.75	31.75
5	Bangladesh	94.19	13.19	45.76	73.10	87.00	334.16	314.57	174.68	209.63	320.30	1666.57	3.72	60.69
6	Morocco	171.64	30.76	13.04	106.53	58.79	232.49	168.38	352.76	146.95	267.70	1549.04	3.45	103.76
7	Tanzania	16.24	39.21	184.42	34.90	83.69	127.61	375.80	147.26	160.45	149.85	1319.44	2.94	84.33
8	Indonesia	47.98	14.65	69.60	30.76	163.39	270.47	46.97	173.56	166.67	247.82	1231.87	2.75	108.70
9	Egypt	111.23	46.73	49.68	55.05	119.31	54.45	86.44	137.37	78.33	228.84	967.44	2.16	32.04
10	Kenya	24.67	7.52	17.73	91.94	45.13	92.65	302.20	60.53	177.07	164.64	984.10	2.19	96.75
	Total sum	2521.33	1735.60	2545.60	3842.77	4251.41	5293.96	5501.59	5740.25	6951.94	6459.93	44,844.38	100.00	12.21
	Mean	28.33	19.28	25.71	40.88	46.21	54.02	53.41	60.42	68.16	64.60			
	SD	55.80	37.40	50.66	105.41	115.14	113.67	121.16	109.69	127.93	92.58			
	Median	6.34	8.25	5.50	9.39	12.55	14.67	10.23	17.80	16.69	31.23			
	IQR (L)	1.73	0.96	0.66	1.24	1.45	1.30	0.89	2.18	1.86	4.80			
	IQR (U)	24.04	19.57	31.46	30.55	40.32	54.44	46.61	79.99	69.80	89.52			

Notes: ODA, official development assistance; SD, standard deviation; IQR, interquartile range.

Iraq also ranked high in ODA per capita for water supply and sanitation, with a commitment above US$ 140 during the 10-year period, in contrast with less than $6 for India, Indonesia, Pakistan, and China (Appendix). The ODA per capita for the water supply and sanitation sector increased in most, but not all, of the 70 Countdown countries during the 10-year period. The largest amount of ODA per capita to the water supply and sanitation sector was committed to Tuvalu, but this was because of its small population. Of the Big 15 countries, no country had more than US$ 100 per capita committed during the 10-year period, except Iraq (Burkina Faso, US$ 48.59; Tanzania, US$ 32.63; Kenya, US$ 26.30; Uganda, US$ 25.58; Niger, US$ 23.62; Afghanistan, US$ 18.52; Angola, US$ 16.39; Malawi, US$ 12.67; Ethiopia, US$ 12.23; Nigeria, US$ 7.01; Indonesia, US$ 5.38; Pakistan, US$ 3.86; India, $US 3.79).

### Number of child deaths to be averted by increasing water and sanitation coverage

If water and sanitation coverage is scaled up to 90% by 2030 with a constant rate of annual increase, under-five deaths could be reduced by 215,935 and 1,300,308 during 2016–2030 in the Countdown countries, respectively (Table 5). In the Big 15 countries, 160,708 and 1,029,466 under-five deaths could be prevented by increasing sanitation and water coverage during this period, respectively. Noticeably, increasing only sanitation coverage could avert 304,768 and 229,065 under-five deaths in India and Nigeria, respectively.

**Table 5.** How many children’s lives could be saved by increasing water and sanitation coverage to 90%?

		U5 deaths to be saved in 2016–2030	
	No. of countries	Water	Sanitation
Total	68	215,935^a^	1,300,308
Big 5	5	92,031^b^	724,175
Big 15	15	160,708^c^	1,029,466
Rank			
1	India	N/A^d^	304,768
2	Nigeria	51,951	229,065
3	DR Congo	40,080	120,595
4	Pakistan	N/A	66,055
5	Afghanistan	12,150	54,543
6	Ethiopia	14,513	52,203
7	Niger	10,784	44,370
8	Mali	6354	36,796
9	Kenya	5877	26,820
10	Burkina Faso	1869	26,609
11	Angola	12,296	26,476
12	Uganda	3388	26,155
13	Indonesia	581	7562
14	Tanzania	865	3757
15	China	N/A	3692

Notes: ^a^57 countries.

^b^2 countries.

^c^12 countries.

^d^(N/A): already over 90% coverage in 2015.

U5, under-five.

## Discussion

Our analysis shows that the inequality of water and sanitation coverage among countries across the world has not been addressed effectively during the past decade, which is a severely pronounced problem in the sanitation sector. The overall increase in water coverage, although it has been articulated as a successful achievement in UN MDG reports [9], has masked an unequal distribution across developing countries.

In previous studies [32,33] of ODA for maternal, newborn, and child health, targeting was found to be improving, although it was not highly targeted to the countries with the highest mortality rates. In our analysis, ODA was not targeted to countries with the highest needs for water and sanitation. The countries with the least coverage persistently received far less ODA per capita than did countries with much higher water and sanitation coverage, suggesting that ODA for water and sanitation is poorly targeted.

Apart from countries with very small populations and rare exceptions such as Iraq, few countries received US$ 100 or more of ODA for water and sanitation per capita per decade; moreover, this amount was much less in the Big 15 countries, where 75% of the global burden of diarrhoea- and pneumonia-related mortality takes place.

Some scholars have argued that the allocation of ODA is motivated by factors other than need, such as political leanings [35,36]. To some extent, this study corroborates that argument by demonstrating that the allocation of ODA to the water and sanitation sector was not determined by need, although we were not able to identify the actual determinants. Further research is necessary to investigate the determinants of ODA allocation for water and sanitation.

Inequalities of water and sanitation coverage among developing countries have not been well accounted for when scaling up interventions in water and sanitation at the global level. Child mortality can be more effectively reduced by increasing water and sanitation coverage in the countries with the least coverage, which bear the largest burden of diarrhoea and pneumonia [29–31].

Increases in sanitation coverage were not in proportion with need among countries. For water coverage, the increase was more pronounced in countries with greater needs, but the association was very weak. It is noteworthy that coverage was much lower in the Big 15 countries. Strikingly, sanitation coverage was severely low in Niger, Tanzania, Burkina Faso, Ethiopia, Mali, and DR Congo, among the Big 15 countries. A high priority should be urgently placed on increasing sanitation coverage, particularly in the Big 15 countries, to reduce child mortality.

Investing in sanitation improvements could prevent a substantial number of child deaths, with an effect much greater than that of improving water coverage. This is intuitively reasonable because there is much more room for improvement in sanitation coverage than in water access, which is a remarkable achievement that has already been accomplished to a great extent across the globe.

With regards to the utility of the Hausman test, a study [37] has demonstrated that there was no need to run the Hausman test in its simulation results.

However, we ran the Hausman test for the following reasons. First, the methodology of this study was based on that of a prior study [32], in which the authors pooled mortality data across countries in two different years and used the Hausman test to select either random effects or fixed effects to assess the trend in ODA. Second, the Hausman test is still frequently used for the selection between random and fixed effects.

A limitation of this study is that we used the amount of ODA commitment, not disbursement, unlike a previous study of aid in the maternal and child health sector [32]. We believe that the committed amount has, to some extent, a meaningful implication since commitment does reflect targeting. However, we recognize that further research should be carried out based on disbursements in the water and sanitation sector.

LiST describes fixed associations between inputs and outputs that will produce the same outputs each time the model is run. The overarching assumption in LiST is that mortality rates and the cause-of-death structure will not change, except in response to changes in the coverage of interventions [38]. Since mortality rates tend to decline over time in many countries, this study might have overestimated the number of deaths averted to some degree.

This study will help the global development community to better understand trends in water and sanitation improvements and the performance of ODA, and provide a benchmark for further research and tracking progress across the globe. We believe that our findings will provide useful insights for global policymakers, international organizations, and donors.

## Conclusion

Although the MDG target in safe drinking water was met in 2010, five years ahead of the original 2015 deadline, it was proved not to have been accompanied by an improvement in equality among countries. The global community have not focused their ODA investment in water and sanitation on the countries of lower income. Particular attention should be given to the key findings of the paper regarding sanitation: an unchanged pro-rich trend across developing countries, poor ODA targeting, and the absence of a faster increase in the countries with the greatest need. The findings demonstrate that improvement of equality should be prioritized in the progress monitoring of SDGs in the water and sanitation sector. The most deprived countries should receive more attention for increases in water and sanitation coverage from the world health community during the SDGs campaign period. A strong political commitment to ODA targeting the countries with the least coverage is needed at the global level.

## Supplementary Material

Appendix

## Paper context

Millennium Development Goal (MDG) 7c for water was declared to have outperformed the goal ahead of the MDGs target year, 2015. However, the inequality of water and sanitation coverage among countries across the world has not been addressed effectively and official development assistance was not targeted to countries with the highest needs for water and sanitation. The most deprived countries should receive more attention for increases in water and sanitation coverage from the world health community.

## Appendix

**Table:**

ODA per capita (Water & Sanitation, USD)												
Rank	country	2001	2002	2003	2004	2005	2006	2007	2008	2009	2010	Total
1	Tuvalu	0.00	0.00	1.77	0.00	0.00	0.00	0.24	0.00	614.29	5.39	621.69
2	Cape Verde	12.73	18.69	3.04	19.46	4.80	47.23	16.17	4.85	63.29	11.92	202.19
3	Lesotho	1.78	3.37	2.06	9.57	26.83	1.56	4.21	79.51	13.77	21.51	164.17
4	Albania	3.71	5.55	11.72	7.54	13.95	4.21	23.33	51.77	11.34	23.96	157.09
5	Iraq	0.02	0.04	1.88	31.64	25.74	23.70	7.16	23.39	14.96	17.76	146.29
6	Maldives	0.21	0.40	0.11	0.06	0.00	14.82	0.38	96.33	1.72	2.38	116.41
7	Guyana	0.00	17.11	0.06	0.00	15.20	14.06	36.35	0.12	15.92	15.87	114.71
8	Djibouti	10.48	10.35	0.94	0.39	0.00	16.56	29.76	13.70	29.23	2.87	114.27
9	Timor-Leste	16.76	1.48	1.21	16.34	11.92	20.83	1.87	3.66	11.11	23.05	108.23
10	Gabon	0.00	0.00	0.02	13.00	0.01	0.00	39.08	0.13	0.13	51.57	103.94
11	Suriname	0.01	11.10	0.83	44.49	0.00	0.18	1.21	0.00	38.80	0.00	96.62
12	Nepal	9.05	0.40	2.07	1.61	1.13	0.81	0.07	0.99	0.43	1.26	91.11
13	Tunisia	2.23	2.42	8.03	15.47	5.05	7.18	12.44	0.19	25.97	8.85	87.84
14	Armenia	2.81	0.76	0.92	18.11	6.88	0.18	13.74	20.32	0.89	13.47	78.10
15	Azerbaijan	0.04	2.42	4.73	2.85	4.15	3.88	0.57	3.45	40.13	3.23	65.46
16	Nicaragua	4.50	0.41	3.22	2.14	1.54	9.48	6.16	17.09	11.81	8.28	64.62
17	Senegal	17.09	1.71	1.16	0.89	5.47	5.11	5.32	2.63	12.26	8.78	60.44
18	Nauru	0.00	0.00	0.00	0.00	17.24	0.00	0.26	21.30	0.81	20.74	60.34
19	Solomon Islands	0.29	0.00	0.07	0.20	1.86	1.68	0.23	0.72	43.53	7.76	56.35
20	Bolivia	1.37	3.40	2.28	5.31	2.19	6.46	4.85	9.09	9.36	11.81	56.12
21	Kiribati	3.14	4.62	0.35	0.00	0.07	0.00	0.00	0.00	0.08	47.20	55.46
22	Mongolia	0.00	3.49	0.42	13.58	0.40	3.03	7.24	5.73	6.41	13.38	53.68
23	Mauritania	0.28	3.73	0.57	10.45	1.24	3.32	5.27	5.63	0.56	20.11	51.16
24	Benin	0.91	0.46	6.02	11.93	8.64	8.63	4.42	2.98	4.42	1.74	50.16
25	Morocco	5.64	1.02	0.43	3.43	1.87	7.54	5.39	11.16	4.59	8.38	49.45
26	Burkina Faso	7.75	3.40	0.31	4.78	1.79	0.20	4.16	6.13	17.89	2.19	48.59
27	Namibia	4.73	3.84	2.67	4.56	2.01	1.12	2.26	3.86	5.61	17.29	47.95
28	Honduras	1.18	0.64	8.17	1.14	3.58	2.10	12.38	7.17	0.83	4.84	42.02
29	Vietnam	3.53	2.50	2.82	3.58	2.31	5.03	2.20	6.22	7.38	4.05	39.62
30	Georgia	1.01	0.96	0.80	0.03	0.04	6.64	0.17	25.86	3.51	0.48	39.50
31	Mozambique	1.64	1.16	0.66	1.04	3.24	3.62	5.05	14.65	3.01	3.41	37.48
32	Ghana	1.93	0.77	1.01	13.20	1.63	4.47	3.81	3.29	0.82	5.89	36.81
33	Mali	0.87	1.50	1.34	2.36	2.90	3.66	3.22	4.92	7.26	6.66	34.69
34	United Republic of Tanzania	0.45	1.08	4.99	0.93	2.18	3.23	9.29	3.47	3.67	3.34	32.63
35	El Salvador	3.97	1.24	1.08	0.87	1.21	0.82	6.36	5.75	6.46	4.80	32.55
36	Zambia	2.26	1.57	1.63	2.43	4.24	4.25	4.65	6.16	2.21	2.39	31.78
37	Yemen	0.19	8.00	0.74	0.65	1.33	3.25	1.51	4.64	9.14	1.51	30.96
38	Gambia	0.15	1.89	0.34	1.82	7.55	2.02	5.98	2.26	0.63	6.36	29.01
39	Papua New Guinea	6.38	0.03	0.02	0.04	2.43	0.03	5.08	0.00	0.01	14.05	28.08
40	Belize	11.04	0.38	0.03	0.00	0.00	0.00	0.00	0.05	0.08	15.43	27.02
41	Kenya	0.79	0.24	0.55	2.75	1.32	2.53	8.05	1.56	4.45	4.06	26.31
42	Comoros	8.06	0.00	0.00	6.68	0.00	1.55	0.00	0.01	1.96	7.93	26.19
43	Uganda	1.93	2.71	3.49	2.23	1.58	3.24	2.54	1.89	3.19	2.78	25.59
44	Chad	0.31	2.02	8.64	1.52	5.43	1.88	0.08	1.40	0.07	3.08	24.44
45	Dominican Republic	0.50	1.15	2.05	1.00	0.20	0.95	0.31	0.50	5.44	11.99	24.12
46	Niger	5.97	0.96	2.62	1.06	1.73	4.86	1.94	2.03	2.02	0.44	23.62
47	Sierra Leone	0.44	0.20	1.66	3.17	0.76	1.85	1.97	0.92	2.75	9.75	23.47
48	Haiti	0.29	0.28	0.66	0.15	0.56	2.76	1.56	0.88	12.27	3.58	22.98
49	Rwanda	0.98	2.61	0.46	1.46	1.69	2.26	2.16	3.59	2.89	4.41	22.52
50	Sao Tome & Principe	4.13	0.16	0.00	0.00	1.12	2.04	3.64	2.40	3.37	4.60	21.45
51	Cambodia	1.94	1.67	2.73	1.40	0.54	1.44	0.39	2.45	4.74	3.10	20.39
52	Peru	0.18	0.70	0.29	1.09	0.45	1.11	0.32	4.76	5.13	6.25	20.28
53	Swaziland	0.00	0.00	5.33	0.20	0.27	0.42	0.76	0.60	0.41	11.99	19.98
54	Liberia	0.00	0.00	0.00	0.10	0.30	0.11	1.72	2.30	1.91	12.95	19.39
55	Burundi	1.32	0.14	1.22	0.49	3.47	3.97	0.09	3.84	1.96	2.26	18.75
56	Laos	1.17	0.23	0.66	0.48	1.91	4.81	6.59	0.35	0.45	1.98	18.63
57	Afghanistan	0.04	1.24	2.18	0.98	0.76	5.41	0.82	3.02	1.96	2.11	18.52
58	Angola	0.71	0.67	2.99	0.27	1.78	0.72	2.98	5.77	0.27	0.25	16.40
59	Kazakhstan	1.23	1.67	12.54	0.09	0.37	0.04	0.02	0.05	0.07	0.01	16.08
60	Guinea-Bissau	0.23	4.99	0.12	0.33	0.10	3.87	0.71	3.29	0.53	1.80	15.98
61	Uzbekistan	0.02	1.11	0.14	0.09	0.36	0.03	1.00	0.12	6.69	5.13	14.69
62	Cameroon	0.30	0.14	0.06	0.17	0.10	2.43	2.13	0.12	3.25	5.84	14.54
63	Guinea	4.91	0.02	0.10	0.58	2.37	1.60	2.60	0.18	0.24	1.64	14.23
64	Ecuador	2.06	0.65	0.58	0.67	0.96	5.15	0.40	0.72	0.28	1.82	13.27
65	Bhutan	0.07	0.00	3.08	0.06	0.39	0.38	0.14	0.23	0.17	8.17	12.69
66	Malawi	0.57	1.18	0.69	0.13	0.33	1.18	3.92	3.06	0.12	1.49	12.67
67	Egypt	1.61	0.66	0.69	0.76	1.61	0.73	1.14	1.69	0.94	2.82	12.66
68	Congo, Rep.	0.00	0.00	4.40	0.23	0.00	0.41	0.00	0.02	5.42	2.18	12.66
69	Ethiopia	0.35	0.31	0.28	1.52	0.25	1.92	2.02	2.47	1.51	1.60	12.23
70	Eritrea	1.12	0.80	2.82	0.13	0.37	1.39	2.83	2.36	0.16	0.15	12.13
71	Guatemala	0.51	0.68	0.42	1.30	1.49	1.58	0.35	0.47	3.20	1.78	11.79
72	Tajikistan	0.60	2.75	0.06	0.81	0.32	2.87	0.41	2.60	0.86	0.46	11.75
73	Equatorial Guinea	0.10	1.98	0.34	7.18	0.37	0.26	1.04	0.00	0.04	0.00	11.31
74	Bangladesh	0.67	0.09	0.31	0.53	0.61	2.14	1.98	1.09	1.29	2.15	10.88
75	Congo, Dem. Rep.	0.02	0.90	0.02	0.02	0.67	0.90	2.86	0.77	3.19	1.20	10.55
76	Zimbabwe	0.10	0.02	0.06	0.01	0.06	0.15	0.13	6.69	1.60	1.32	10.14
77	Madagascar	1.24	0.41	0.28	0.76	0.45	4.37	0.28	0.59	0.19	0.47	9.04
78	Central African Rep.	0.06	0.00	0.00	0.00	0.00	0.33	2.65	0.02	2.48	3.41	8.95
79	Marshall Islands	0.00	0.00	0.03	0.30	0.15	2.96	0.46	0.00	1.21	3.78	8.88
80	South Africa	0.14	0.44	1.06	1.43	0.35	1.20	3.14	0.16	0.15	0.05	8.12
81	Turkey	0.92	0.00	0.00	0.00	0.63	0.00	0.03	0.32	4.07	1.23	7.21
82	Nigeria	0.51	0.43	0.15	1.67	1.55	0.71	0.81	0.57	0.04	0.56	7.01
83	Cote d'Ivoire	0.92	0.01	0.03	0.00	0.01	0.02	0.11	2.88	0.01	2.38	6.37
84	Algeria	0.40	1.04	1.16	0.21	1.07	0.10	0.10	0.05	0.55	1.27	5.93
85	Togo	0.37	0.00	0.14	1.51	0.02	0.09	0.04	0.34	2.59	0.40	5.49
86	Indonesia	0.22	0.07	0.32	0.14	0.73	1.18	0.20	0.76	0.72	1.03	5.39
87	Pakistan	0.04	0.08	0.38	0.14	0.41	0.16	0.17	0.53	0.75	1.20	3.86
88	India	0.24	0.15	0.26	0.09	0.53	0.56	0.78	0.26	0.67	0.24	3.79
89	Brazil	0.02	0.00	0.18	1.08	0.11	0.04	0.02	0.02	0.18	1.26	2.90
90	Somalia	0.21	0.41	0.30	0.39	0.08	0.29	0.23	0.46	0.41	0.06	2.85
91	Paraguay	0.00	0.00	0.02	0.34	0.04	0.09	0.06	0.21	1.44	0.35	2.54
92	Micronesia, Fed. States	0.00	0.00	0.01	0.00	0.00	0.52	0.02	0.00	0.22	1.57	2.35
93	China	0.16	0.16	0.17	0.13	0.42	0.19	0.42	0.07	0.12	0.12	1.96
94	Philippines	0.19	0.18	0.44	0.03	0.15	0.17	0.16	0.24	0.25	0.14	1.95
95	Botswana	0.00	0.02	1.33	0.00	0.01	0.34	0.06	0.04	0.00	0.06	1.85
96	Mexico	0.01	0.00	0.01	0.01	0.68	0.52	0.07	0.02	0.12	0.28	1.71
97	Myanmar	0.12	0.02	0.06	0.02	0.01	0.03	0.05	0.08	0.07	0.22	0.66
98	Oman	0.00	0.00	0.01	0.01	0.01	0.02	0.06	0.00	0.13	0.09	0.34
99	Iran	0.00	0.00	0.01	0.04	0.04	0.04	0.03	0.02	0.02	0.02	0.22
100	Korea, Dem. Rep.	0.03	0.01	0.02	0.00	0.02	0.01	0.04	0.05	0.01	0.03	0.21
101	Turkmenistan	0.00	0.07	0.00	0.00	0.00	0.01	0.01	0.00	0.01	0.00	0.11
102	Saudi Arabia	0.00	0.00	0.00	0.00	0.00	0.00	0.02	0.00	0.00	0.00	0.03
103.00	Libya	0.00	0.00	0.00	0.00	0.00	0.00	0.00	0.00	0.00	0.00	0.01
